# PD79 Cost Heterogeneity Across Health Sectors And Its Impact On Coverage Decisions for Metastatic Prostate Cancer Treatment In Argentina

**DOI:** 10.1017/S0266462325102067

**Published:** 2025-12-29

**Authors:** Jorge Elgart, Mariana Glancszpigel, Gonzalo Guiñazú, Juan De Bonis, Silvana De Cicco, Lucila Rey-Ares

## Abstract

**Introduction:**

Argentina’s healthcare system is fragmented, comprising public, private, and social security sectors. The guidance for incorporating a new health technology into the healthcare system suggests a single threshold value (0.016% of total health expenditure). This study analyzed the influence of cost heterogeneity on budget impact using the reimbursement of talazoparib for the treatment of metastatic prostate cancer as an example.

**Methods:**

A budget impact analysis (BIA) was adapted for the National Institute of Social Services for Retirees and Pensioners and SURGE (union social security sector) perspectives. To be able to estimate the impact of medical costs, other parameters such as population and market share were fixed. Drug costs were estimated using reimbursement values, while the costs of healthcare resources and management of adverse events were obtained from the 3Eff SA cost database (3val), which is based on tariffs of institutions within the subsectors of the Argentine healthcare system.

**Results:**

For direct medical resources (medication, medical consultations, laboratory tests, etc.) associated with metastatic prostate cancer treatment, cost heterogeneity across health sectors was 79 percent. The lowest variability was observed in laboratory tests (18%), while the highest was in medical consultations (310%). The cost of managing adverse events varied by 38 percent across sectors, with the highest variability in managing fatigue (118%) and the lowest in thrombocytopenia (2.4%). As a result, the average annual budget impact varied across sectors by just over 15 percent.

**Conclusions:**

Our results showed a significant heterogeneity in costs among Argentina’s healthcare subsectors. This heterogeneity might lead to different reimbursement decisions based on the BIA results. Variability in costs underscores the need for sector-specific guidelines or thresholds for reimbursement decisions to ensure equitable and efficient resource allocation.

